# Advances in Catalysts for Hydrogen Production: A Comprehensive Review of Materials and Mechanisms

**DOI:** 10.3390/nano15040256

**Published:** 2025-02-08

**Authors:** Niraj Kumar, Radhamanohar Aepuru, Seul-Yi Lee, Soo-Jin Park

**Affiliations:** 1 Department of Chemistry, Inha University, Incheon 22212, Republic of Korea; niraj@inha.ac.kr; 2 Departamento de Mecánica, Facultad de Ingeniería, Universidad Tecnológica Metropolitana, Santiago 7800002, Chile; raepuru@utem.cl; 3 Department of Mechanical Engineering, College of Engineering, Kyung Hee University, Yongin 17104, Republic of Korea; 4 Department of Advanced Materials Engineering for Information and Electronics, Kyung Hee University, Yongin 17104, Republic of Korea

**Keywords:** hydrogen production, catalysts, nanostructured materials, photocatalysis, sustainable energy

## Abstract

This review explores the recent advancements in catalyst technology for hydrogen production, emphasizing the role of catalysts in efficient and sustainable hydrogen generation. This involves a comprehensive analysis of various catalyst materials, including noble metals, transition metals, carbon-based nanomaterials, and metal–organic frameworks, along with their mechanisms and performance outcomes. Major findings reveal that while noble metal catalysts, such as platinum and iridium, exhibit exceptional activity, their high cost and scarcity necessitate the exploration of alternative materials. Transition metal catalysts and single-atom catalysts have emerged as promising substitutes, demonstrating their potential for enhancing catalytic efficiency and stability. These findings underscore the importance of interdisciplinary approaches to catalyst design, which can lead to scalable and economically viable hydrogen production systems. The review concludes that ongoing research should focus on addressing challenges related to catalyst stability, scalability, and the integration of renewable energy sources, paving the way for a sustainable hydrogen economy. By fostering innovation in catalyst development, this work aims to contribute to the transition towards cleaner energy solutions and a more resilient energy future.

## 1. Introduction

Catalysts are integral to the efficient production of hydrogen, a versatile and clean energy carrier with immense potential to address global energy demands and mitigate the impacts of climate change [1,2,3,4]. The pursuit of cost-effective and highly efficient catalysts has become the cornerstone of sustainable energy research, driven by the growing urgency to transition from fossil fuels [5,6]. Recent advancements in catalytic technology have significantly enhanced hydrogen production, offering improvements in efficiency, scalability, and environmental sustainability [7]. In general, the production of hydrogen is achieved through various methods, each defining a specific type of hydrogen. For instance, depending on the amount of the emitted CO_2_ and renewability, the type of hydrogen is named as gray, blue and green [8]. Among these, green hydrogen has significant importance due to its cleaner and more sustainable alternative energy during the hydrogen production through various methods such as biogas re-forming, biochemical conversion, and the photocatalysis and electrolysis of water, which produce zero CO_2_ emissions and utilize renewable material sources as feedstock. The efficient use of renewable resources promotes environmental sustainability, helps mitigate climate change, and reduces the reliance on fossil fuels [9]. This review provides a detailed exploration of the latest developments in catalyst materials and mechanisms and emphasizes their implications for advancing hydrogen production technologies. Emerging research has identified a diverse range of materials and approaches for enhancing the catalytic performance. Among these, nanomaterials have demonstrated exceptional promise owing to their high surface area and tailored reactivity, which enable superior catalytic activity and stability [10,11]. Transition metals and metal-free catalysts derived from earth-abundant resources are being increasingly investigated as cost-effective alternatives to traditional precious metals [12]. Multifunctional catalysts capable of integrating processes, such as water splitting and carbon dioxide reduction, have garnered attention for their potential to streamline hydrogen production and improve the overall system efficiency [13,14]. Bio-inspired catalysts that mimic natural photosynthetic mechanisms have also shown significant potential, particularly for achieving high selectivity and activity under mild reaction conditions [15,16]. These developments are supported by advances in characterization techniques, such as spectroscopic and microscopic methods, which facilitate the real-time monitoring of catalytic reactions [17,18]. This capability has provided critical insights into reaction mechanisms and durability, enabling the design of more effective catalysts.

Despite these advancements, significant challenges remain in catalyst development. Understanding the structure–function relationships that dictate catalytic behavior is a persistent obstacle, as these relationships often lack comprehensive elucidation [19,20]. Moreover, translating laboratory-scale innovations into industrial applications requires addressing issues related to long-term stability, mass transport, and thermal management in large-scale systems [21]. Scalability also remains a concern, because many high-performance catalysts rely on complex or resource-intensive synthesis methods [22,23]. The integration of advanced catalysts into full-scale hydrogen production systems requires multidisciplinary approaches that encompass material science, engineering, and system optimization [24]. This field is poised to benefit from a combination of innovative research directions. The design of hierarchical and hybrid catalyst architectures can enhance catalytic efficiency by optimizing the active sites and improving reactant accessibility [25]. Computational modeling and machine-learning techniques are expected to play a growing role in predicting catalyst behavior and accelerating the discovery of novel materials [26,27]. Additionally, efforts to integrate catalytic systems with renewable energy sources such as solar or wind power could further enhance the sustainability of hydrogen production [28,29,30]. Advances in modular reactor design and decentralized production systems are anticipated to facilitate the adoption of hydrogen as a clean energy carrier for diverse applications.

**Figure 1:**
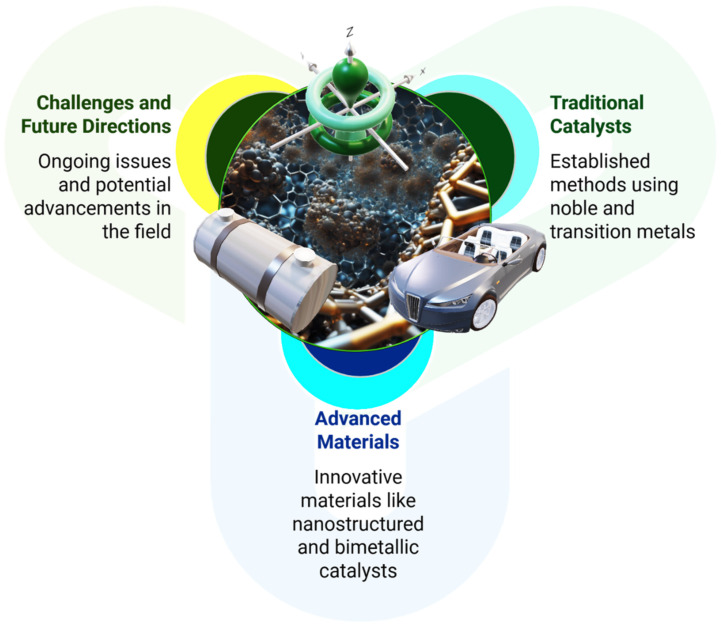
Trends in hydrogen production catalysis.

This review presents the recent advancements in catalyst technology for hydrogen production and highlights the progress, challenges, and opportunities in this rapidly evolving field. The exploration of a broad spectrum of catalytic materials, mechanisms, and performance outcomes provides valuable insights for researchers, engineers, and policymakers dedicated to the development of sustainable energy solutions. These findings underscore the critical role of innovative catalyst design in realizing the full potential of hydrogen as the cornerstone of a cleaner and more sustainable energy future. Through continued interdisciplinary collaboration and investment in research, the field of catalysis is well positioned to address the remaining challenges and unlock new possibilities for hydrogen production technologies, paving the way for a transformative energy landscape. Figure 1, a visual representation of the emerging materials and technologies, presents an overview of various advanced catalysts, including catalysts for hydrogen production. This illustration highlights the innovative materials used in hydrogen production for sustainable energy applications.

## 2. Mechanisms of Hydrogen Evolution and Catalytic Performance

### 2.1. Thermochemical Mechanisms

Hydrogen evolution through thermochemical processes, particularly water splitting, has become a critical focus in the development of sustainable energy solutions. Over the years, platinum (Pt) has served as a benchmark catalyst because of its activity in the hydrogen evolution reaction (HER). However, their high cost and limited availability necessitate the search for affordable alternatives. This shift has sparked significant advancements, particularly in understanding fundamental reaction mechanisms, such as the Volmer–Heyrovsky–Tafel pathways. These insights provide a foundation for optimizing catalytic processes and introducing innovative materials with promising results. Among the recent breakthroughs, ruthenium (Ru)-based heterostructure catalysts have emerged as effective and economical alternatives to Pt. Through innovative structural engineering and synthetic strategies, these catalysts maintain high catalytic activity and exhibit remarkable stability. Similarly, considerable progress has been made in the development of platinum-free electrocatalysts, including transition metal phosphides, carbides, and nitrides. Their capacity to achieve efficient HER kinetics while reducing cost highlights their potential for large-scale applications [31].

Advances in seawater electrolysis have opened new avenues for hydrogen production. Transition metal phosphides (TMPs), for instance, present high potential for this process by addressing challenges such as electrode corrosion and chlorine evolution. With enhanced structural engineering and interface modifications, TMPs have proven scalable and environmentally friendly. Additionally, thermochemical routes such as biomass pyrolysis using microwave-assisted processes have emerged as sustainable and viable options for hydrogen generation [32,33]. Despite these advancements, challenges persist regarding the stability of non-precious metal catalysts under harsh conditions and the scalability of laboratory findings for industrial applications. Furthermore, issues related to selectivity in complex systems, as observed in seawater electrolysis, add another layer of complexity to the field. Overcoming these hurdles will require a multifaceted approach that encompasses the development of innovative materials, a deeper mechanistic understanding, and strategic integration with renewable energy sources. Looking ahead, the future of the hydrogen evolution technology is promising. Research is poised to transform this field by exploring single-atom catalysts for designing hybrid systems and optimizing lattice oxygen-mediated mechanisms. Integrating these advancements with solar and wind energies will further enhance their sustainability and scalability. With continued innovation, thermochemical hydrogen evolution has the potential to efficiently and sustainably meet global energy demands.

### 2.2. Electrochemical Hydrogen Evolution Reaction

Advancements in electrochemical hydrogen evolution reactions have facilitated sustainable hydrogen production. Historically, noble metals such as platinum have been regarded as the gold standard owing to their efficiency; however, their high cost and scarcity have prompted the exploration of alternative materials. This search has been guided by an understanding of the fundamental mechanisms of the HER and the Volmer, Heyrovsky, and Tafel steps, which dictate the formation and evolution of hydrogen atoms on catalytic surfaces.

A significant breakthrough has been the development of platinum-free catalysts such as molybdenum carbide (Mo_2_C) nanocrystals, which demonstrate excellent activity in various pH environments [34]. Similarly, self-supported catalysts such as layered double hydroxides (LDHs) exhibit improved stability and reduced energy barriers for the HER [35]. Heterostructural engineering, exemplified by ruthenium-based catalysts, has advanced electron transfer rates to address kinetic limitations. Because of their conductivity and adaptable surface chemistry, MXene-based nanohybrids exhibit enhanced HER capabilities when combined with transition metals [36]. Moreover, defect engineering, which involves the introduction of atomic-level imperfections in MoS_2_, has emerged as a significant advancement by enhancing active-site exposure and reducing energy demands [37]. Despite these advancements, several challenges remain to be overcome. The durability under extreme conditions, incomplete understanding of atomic-scale HER mechanisms, and high costs associated with scaling these materials have hindered progress. Additionally, achieving a low energy input while maintaining high Faradaic efficiency is a considerable hurdle. The integration of computational tools, such as density functional theory (DFT), with experimental approaches can expedite the discovery of optimized catalysts. Hybrid materials that combine strengths from different classes, innovative electrode designs to enhance mass transport, and environmentally friendly synthesis methods hold promise for addressing the current limitations. In situ characterization techniques are crucial for obtaining real-time insights into material behavior and reaction pathways. As the development of cost-effective and efficient HER catalysts continues, interdisciplinary research combining material science, chemistry, and engineering is essential to unlock the full potential of hydrogen as a clean energy source.

### 2.3. Photocatalytic Mechanisms

The photocatalytic hydrogen evolution reaction represents a transformative approach to achieving sustainable energy solutions by converting sunlight into clean hydrogen fuel through water splitting. Originating in the 1970s with foundational work on titanium dioxide (TiO_2_) photocatalysis, the field has continually evolved to emphasize the critical role of semiconductors in driving the HER. Researchers have refined material designs, explored the role of co-catalysts, and unraveled reaction mechanisms, all of which are aimed at enhancing the efficiency while minimizing costs. This progression highlights the ingenuity and resilience of scientific inquiry in addressing the global energy challenges. Innovations in material science have significantly impacted the efficiency and feasibility of the HER. Among these advancements, ruthenium-based catalysts stand out for their remarkable catalytic activity and cost-effectiveness, making them viable alternatives to expensive platinum-based options. Ruthenium catalysts, optimized through heterostructure design, offer an ideal balance between activity and stability. Complementing this, ultrathin two-dimensional materials, such as MoS_2_ and WS_2_, have garnered attention owing to their tunable band gaps and exceptional electron transport properties. By enhancing the density of the active sites and modifying the electronic structures, these materials have demonstrated significantly improved HER performance [38]. Another breakthrough involves single-atom catalysts (SACs), such as Pt and Co, embedded in semiconductor matrices, which maximize atomic efficiency and provide unique electronic configurations for superior catalysis [38]. A deeper understanding of the HER mechanism is essential. Photocatalysts operate by absorbing light to generate electron–hole pairs, driving the catalytic reaction [39]. Advances in areas such as piezoelectric effects and spintronics have enabled more effective charge separation and minimized recombination losses, thereby enhancing overall efficiency. Techniques such as piezo-stimulated polarization have further pushed the boundaries of the photocatalytic performance [40].

**Figure 2:**
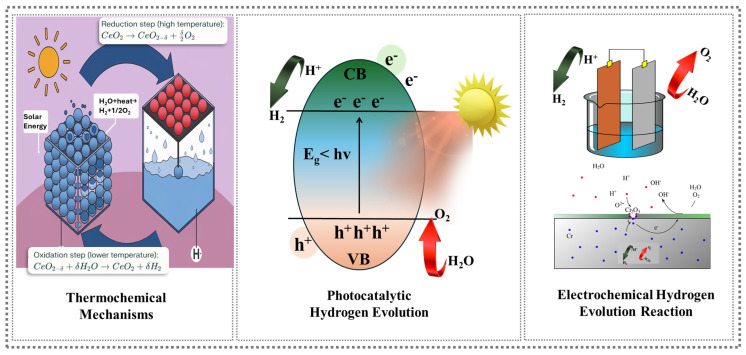
Mechanisms of hydrogen production.

Progress in the HER is also due to experimental innovations that refine catalyst properties and improve system performance. For example, heterojunction engineering, such as combining molybdenum carbide (MO_2_C) with semiconductors, enhances light absorption and charge transfer, rendering these systems highly efficient (Zhou et al.) [41]. Additionally, synthetic strategies, such as chemical vapor deposition (CVD) and hybridization techniques, have provided precise control over the material properties, enabling better catalytic behavior and efficiency. Despite notable advancements, significant challenges remain in the widespread adoption of photocatalytic HER technology. One major issue is efficiency and stability, as many photocatalysts exhibit low quantum efficiencies and degrade under extended usage. Material costs also remain a barrier, particularly when scaling up production while maintaining high performance. Finally, the complexity of the reaction mechanisms poses challenges, requiring sophisticated characterization methods to unravel the intricate interplay between light, catalyst surfaces, and reaction intermediates. The ongoing debate within the research community reflects the differing priorities in and approaches to the development of the HER. While some researchers advocate for high-surface-area materials such as 2D TMDs, owing to their exceptional electronic and structural properties, others emphasize the potential of single-atom catalysts for achieving unparalleled atomic efficiency. This divergence underscores the need for a balanced approach that integrates cost, scalability, and catalytic performance. Looking ahead, several pathways promise to advance the field of the photocatalytic HER. First, the development of earth-abundant, low-cost materials with tailored properties and enhanced stability is critical for overcoming the current material limitations. Secondly, integrating photocatalysis with renewable energy systems could enable practical applications in hydrogen production and storage, bridging the gap between research and real-world applications. Finally, leveraging computational modeling, such as machine learning and quantum simulations, offers a powerful tool for predicting and designing next-generation catalysts with unprecedented precision. The schematic representation in Figure 2 illustrates the thermochemical and electrochemical hydrogen evolution reactions (HERs) and photocatalytic mechanisms involved in hydrogen production.

The evolution of the photocatalytic HER demonstrates the potential of innovative science to address the global energy challenges. Although recent advancements in materials and experimental techniques have driven significant progress, overcoming the hurdles related to efficiency, stability, and scalability is crucial for widespread adoption. By fostering synergies between theoretical insights and experimental breakthroughs, this field is poised to unlock the full potential of photocatalysis, paving the way for a sustainable, hydrogen-based energy future. Despite notable advancements, significant challenges remain in the widespread adoption of photocatalytic HER technology. One major issue is the efficiency and stability, as many photocatalysts exhibit low quantum efficiencies and degrade under extended usage.

### 2.4. Key Performance Indicators (Activity, Stability, Selectivity)

Advancing the HER is a key area of renewable energy research that addresses the challenges in energy conversion and storage. Initially reliant on noble metal catalysts such as platinum for their high activity and stability, the field is now shifting towards sustainable alternatives. This focus has expanded to the development of earth-abundant materials that compete in activity, stability, and selectivity across various media, highlighting the need to optimize key performance indicators (KPIs) for practical applications. Recent advancements have led to innovative approaches for the development of HER catalysts. Defect engineering has been transformative, with Mn-oxide/Fe–Ni sulfide hybrids enhancing their activity through abundant active sites at their interfaces [42]. Similarly, synergistic designs, such as Mo_2_C/CeO_2_ nanoparticles on N-doped carbon, combine to accelerate charge transport and improve durability [43]. Innovations, including morphology-controlled cobalt phosphide nanostructures and operando spectroscopy, further enhance the catalytic performance [44,45].

Despite progress, challenges, such as scalability and cost, hinder large-scale adoption, whereas long-term durability across varying pH conditions poses significant obstacles. The transient transformations at catalytic interfaces necessitate a deeper mechanistic understanding, particularly as activity–selectivity trade-offs complicate practical applications. Researchers have debated topics such as defect engineering, optimal material choices, and the role of transient states in reflecting the evolution of the field. The integration of computational and experimental methodologies offers a promising solution. High-throughput screening powered by machine learning can expedite the identification of optimal catalyst compositions, whereas advanced operando techniques are crucial for uncovering catalytic mechanisms. Multifunctional catalysts that support both the HER and the oxygen evolution reaction (OER) can streamline water-splitting systems, and hybrid approaches combining photocatalysis with electrochemistry may enhance energy efficiency [46,47]. Innovations in protective coatings and self-healing materials promise to improve durability without sacrificing performance. Table 1 outlines the KPIs in terms of activity, stability, and selectivity for HER mechanisms and catalytic performance based on recent research findings. The development of HER catalysts is a crucial aspect of material science and energy technology. Although significant progress has been made in designing efficient catalysts, addressing the economic and mechanistic challenges is essential for unlocking their full potential. A multidisciplinary approach that leverages cutting-edge technologies is likely to pave the way for accessible clean hydrogen energy.

## 3. Traditional Catalysts for Hydrogen Production

Hydrogen production relies on various catalysts, including noble metals, such as platinum, palladium, and rhodium, as well as non-noble metals, such as nickel and copper [54,55]. To enhance their stability and effectiveness, these catalysts are often supported on materials, such as carbon, metal oxides, or zeolites [54,56]. Current research has focused on developing cost-effective and sustainable alternatives to traditional catalysts, particularly high-entropy materials comprising five or more uniformly distributed metallic components.

### 3.1. Noble Metal Catalysts

Noble metal catalysts, including Pt, Pd, Ru, Rh, and Ir, are important for hydrogen production technologies, particularly for electrocatalytic and photocatalytic water splitting. Their unique electronic structures and catalytic properties have established them as standards for the hydrogen evolution reaction (HER) and oxygen evolution reaction (OER) in water electrolysis [57]. However, their high cost and limited availability have spurred research aimed at optimizing their performance and developing cost-effective alternatives [58]. Recent advancements in noble metal catalysts have focused on enhancing their efficiency and sustainability through innovative strategies. Structural engineering and microenvironment modulation seek to optimize catalytic site design and reactivity correlations. Techniques such as crystal phase modulation, alloying, and nanoscale structuring enhance electron transfer and catalytic performance [59]. Single-atom catalysts (SACs) provide increased active sites and improved stability owing to their unique metal-support interactions, which represents a promising avenue for maximizing noble metal utilization [60]. Furthermore, high-throughput computational methods, including density functional theory (DFT) and machine learning, facilitate the predictive design of catalysts, enabling researchers to explore new alloys and catalytic sites and fine-tune electronic properties.

Innovations in SACs, hybrid catalysts, and computational design present promising pathways, and further research is essential to unlock the full potential of noble metals in supporting global hydrogen production goals.

**Figure 3:**
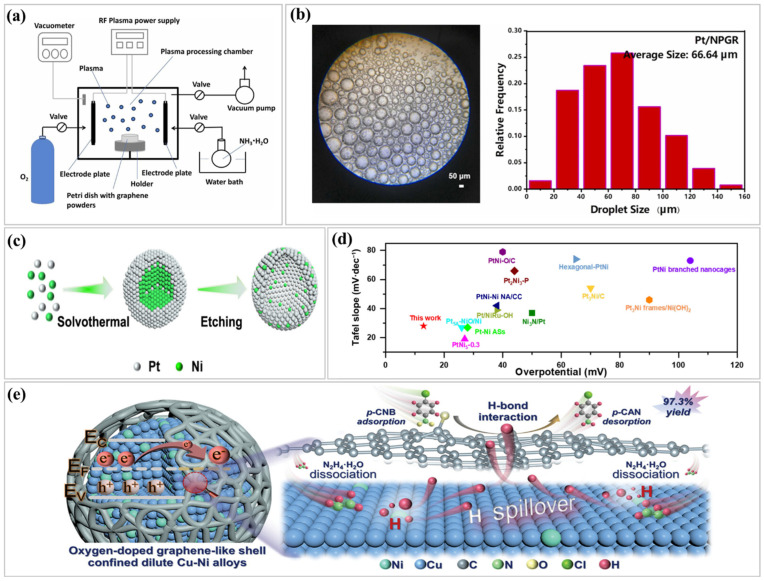
(**a**) Synthesis of N- and O-doped graphene via RF cold plasma treatment; (**b**) optical image of Pt/NPGR emulsions and droplet distribution, reproduced with permission [61], Copyright © 2023, Elsevier B.V.; (**c**) schematic of structural evolution in PtNi-mixed catalyst during acid etching; (**d**) comparison of HER performance in PtNi catalysts, reproduced with permission [62], Copyright © 2024, Springer nature, and (**e**) proposed mechanism for enhanced hydrogenation in CuNi_0.05_@OC Catalyst, reproduced with permission [63], Copyright © 2024, Wiley.

For example, Wang et al. [61] synthesized nitrogen-doped graphene with an amphiphilic surface using radio-frequency cold plasma with ammonia as the plasma source. Nitrogen-doped graphene (NPGR) was used as a support for a Pt nanocatalyst (Pt/NPGR) in the aqueous-phase hydrogenation of cinnamaldehyde. Nitrogen- and oxygen-doped graphene samples were synthesized using a cold plasma treatment device with a 13.56 MHz radio frequency generator in a vacuum chamber (Figure 3a). The NPGR-supported platinum nanocatalyst showed high catalytic performance owing to the enhanced surface properties of nitrogen-doped graphene, such as increased anchoring sites for platinum nanoparticles and improved metal–support interactions. The Pt/NPGR nanocatalysts exhibited enhanced catalytic performance in the aqueous-phase hydrogenation of cinnamaldehyde facilitated by a Pickering emulsion system (Figure 3b). This study provides a green and efficient method for synthesizing graphene-based nanocatalysts with various surface properties. Another important advance involves the synthesis of noble metal alloys and hybrid structures. By combining noble metals with transition metals or non-metal elements, scientists can modify the electronic properties of the catalyst to optimize hydrogen adsorption energy (ΔGᴴ). Cheng et al. [62] showcased this approach using a PtNi alloy (Pt_3_Ni_2_NW_s_-S/C), which outperformed pure Pt catalysts for the HER because of the synergistic interactions between the Pt and Ni atoms and the formation of active Pt(111) facets. The schematic model illustrates the structural changes in the PtNi-mixed catalyst during acid etching (Figure 3c). Figure 3d compares the HER performance of various PtNi catalysts, highlighting the superior catalytic efficiency of the synthesized PtNi-mixed catalyst. This alloying strategy enables the precise tuning of the catalytic properties, resulting in notable enhancements in activity and efficiency. Carbon-based materials such as graphene and carbon nanotubes have garnered attention owing to their high surface area and excellent electrical conductivity. For instance, Yuan et al. [63] developed a graphene chainmail-shelled dilute Cu-Ni alloy catalyst for selective aqueous-phase catalytic hydrogenation. The CuNi_0.05_@OC catalyst exhibited excellent activity, selectivity, and durability for various hydrogenation reactions. Figure 3e illustrates the proposed mechanism for the improved catalytic transfer hydrogenation performance of p-CNB to p-CAN with the CuNi0.05@OC catalyst, emphasizing key factors such as metal–support interaction, isolated Ni sites, alloy and strain effects, and optimized oxygen-doped graphene layers. The three-layered graphene chain encapsulated the dilute Cu-Ni alloy and provided robust protection. This study offers a simple and environmentally friendly strategy for the development of practical heterogeneous catalysts that combine the synergistic effects of dilute alloys and functional carbon wrapping.

Despite these advancements, challenges remain in the practical deployment of noble metal catalysts. Their high cost and scarcity are significant barriers to large-scale applications, whereas issues such as catalyst degradation and restructuring during prolonged operation impede long-term stability [64]. Additionally, there is an ongoing debate regarding whether non-noble metal alternatives, such as MoS_2_, WS_2_, and Fe-Ni alloys, can achieve comparable performance at a fraction of the cost, raising questions about the future of noble metal-based systems [38]. To address these challenges, researchers are investigating hybrid catalysts that combine noble metals with transition metals or carbon-based materials to reduce cost without compromising efficiency [65]. Stability enhancement strategies, including surface modifications and protective coatings, are essential for prolonging the lifespan of catalysts. Moreover, scalable and sustainable production methods, along with advanced in situ characterization techniques, are crucial for unraveling the reaction mechanisms, understanding catalyst degradation, and optimizing commercial viability. While noble metal catalysts remain central to hydrogen production technologies because of their unparalleled catalytic efficiency, their future depends on overcoming the challenges related to cost, stability, and sustainability.

### 3.2. Transition Metal Catalysts

**Figure 4:**
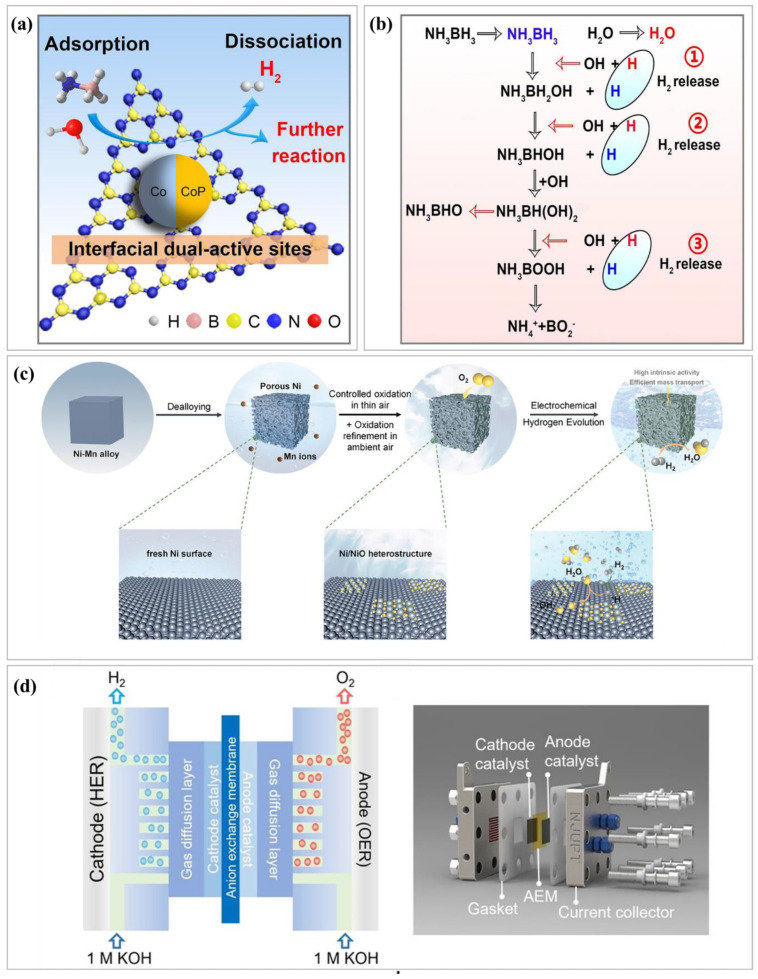
(**a**,**b**) Proposed mechanism of hydrogen generation from NH_3_BH_3_ hydrolysis on CoP-Co/CN-I [70], Copyright © 2024, Elsevier B.V, (**c**) fabrication process of nanoporous Ni/NiO heterostructure catalysts [71], Copyright © 2024, Wiley, and (**d**) Flow-Type AEM Electrolyzer with W_2_S_3_ NSs Cathode for Hydrogen Production [72], Copyright © 2024, Wiley.

Transition metal catalysts are emerging as a cornerstone in the development of efficient and sustainable hydrogen production technologies, offering a viable alternative to conventional fossil fuel-based energy systems [66]. This section delves into recent advancements, persistent challenges, and prospective directions in the field of transformation. Hydrogen production via water splitting has been recognized as a promising solution to the global energy crisis. Historically, noble metals such as platinum have dominated this domain because of their exceptional catalytic activity. However, their scarcity and high costs have driven research towards more abundant and cost-effective alternatives, particularly transition metal-based catalysts. These catalysts, characterized by their versatility and tunable properties, have demonstrated significant potential for the HER [67,68]. Substantial progress has been made in the development of efficient transition metal catalysts for the HER. Among these, transition-metal nitrides (TMNs) have gained considerable attention. The incorporation of nitrogen atoms alters the d-band structure of transition metals, effectively shrinking the d-band width and building active sites with electronic properties similar to those of Pt. This modification enhances the catalytic activity, thermal stability, and electronic conductivity of TMNs, making them a promising class of materials [69]. Zhang et al. [70] introduced a novel approach to hydrogen generation by designing a CoP/Co heterojunction on porous g-C_3_N_4_ nanosheets. The mechanism, in Figure 4a, and catalytic hydrolysis, in Figure 4b, of NH_3_BH_3_ on CoP-Co/CN-I involves sequential OH*-mediated attacks, leading to B-H and O-H bond activation, hydrogen release, and transformation into NH_4_^+^ and BO_2_^−^. This catalyst was developed through pyrolysis- and phosphorus-inducing strategies, resulting in highly exposed active sites, optimized hydrogen and water absorption free energies, and enhanced catalytic activities. The catalyst achieved a turnover frequency of 26 min^−1^ at room temperature, positioning it among the leading non-precious metal phosphides. Its catalytic performance remained stable even after five cycles, indicating significant advancement in non-noble metal catalysts for industrial applications in heterogeneous catalysis. Building on advancements in hydrogen production, Qiao et al. [71] developed a nanoporous Ni/NiO heterostructure catalyst using a two-step process. The schematic illustration in Figure 4c includes the environmentally sustainable fabrication process for nanoporous Ni/NiO heterostructure catalysts, involving the dealloying of Ni_30_Mn_70_ sheets in (NH_4_)_2_SO_4_ solution, followed by controlled oxidation to form a protective NiO layer. First, chemical dealloying creates a 3D nanoporous structure, followed by controlled oxidation to generate uniform NiO layers on the nickel matrix. This design demonstrates exceptional performance in the hydrogen evolution reaction (HER) and remarkable stability in alkaline environments, opening new possibilities for advanced metal/oxide heterostructured catalysts for energy applications. Xie et al. [72] engineered a metallic tetra-coordinated W_2_S_3_ crystal using a precise stoichiometric growth strategy. A flow-type AEM electrolyzer was assembled using W_2_S_3_ NSs as the cathode and commercial IrO_2_ as the anode to evaluate its feasibility for large-scale hydrogen production (Figure 4d). This crystal exhibits outstanding bifunctionality and stability as a cathode catalyst for hydrogen production in both acidic and alkaline electrolytes. The W_2_S_3_ crystal demonstrated Pt-like HER activity, characterized by low overpotentials and Tafel slopes, underscoring its potential as a high-performance non-noble metal electrocatalyst. This work highlights the promise of sustainable hydrogen production technologies that rely on innovative nonprecious metal designs. To better understand the structure–activity relationships of transition metal catalysts, researchers have employed advanced experimental techniques. X-ray absorption spectroscopy (XAS) and in situ/operando methods are particularly valuable for elucidating the local electronic structures and oxidation states of catalysts under working conditions. These studies revealed that the active sites undergo dynamic changes during the HER, challenging earlier models that treated these sites as static entities. SACs represent another innovative approach in which individual transition metal atoms are dispersed on suitable supports. This strategy maximizes the metal utilization and allows for the precise control of the electronic properties of the active sites, leading to remarkable catalytic efficiencies with minimal material consumption. Despite these advancements, several challenges remain. The stability of the catalysts under harsh operating conditions, particularly in alkaline media, remains a critical concern. Although some studies have reported excellent durability, others have highlighted the gradual degradation, underscoring the need for more robust materials. Additionally, the exact nature of the active sites in transition metal catalysts continues to be debated, with divergent views on the roles of the metal centers, ligands, and support materials.

Further research in this field has immense potential in this regard. The rational design of multi-metallic systems can leverage synergistic interactions to enhance both catalytic activity and stability. Advances in in situ characterization techniques, particularly those enabling real-time atomic-scale observations, could provide deeper insights into reaction mechanisms. Computational methods including machine learning and high-throughput screening have been proposed to accelerate the discovery of novel catalysts. Moreover, integrating transition metal catalysts with renewable energy inputs to enable efficient hydrogen production during intermittent energy supply is a critical step toward sustainability. Finally, addressing scalability challenges for industrial applications is essential for transitioning from laboratory-scale innovation to practical deployment. Transition metal catalysts for hydrogen production represent a rapidly advancing field with the potential to revolutionize energy systems. Although significant progress has been made in enhancing catalytic performance and understanding fundamental mechanisms, overcoming the current limitations will require sustained interdisciplinary research. By addressing these challenges, transition metal catalysts can play a critical role in enabling scalable and sustainable hydrogen production, thereby contributing to a cleaner and more resilient energy future.

## 4. Advanced Materials for Hydrogen Production Catalysts

Table 2 highlights the critical physical, chemical, catalytic, environmental, structural, compositional, and hydrogen interaction properties of advanced materials, such as carbon-based nanomaterials, metal oxides, single-atom catalysts, bimetallic/multi-metallic catalysts, and MOFs.

### 4.1. Nanostructured Catalysts

#### 4.1.1. Carbon-Based Nanomaterials

Carbon-based nanomaterials (CBNs) have garnered significant attention as potential catalysts for hydrogen production, offering a sustainable and environmentally friendly alternative to conventional energy sources [78]. Characterized by their high surface area, excellent electrical conductivity, and chemical stability, these materials are at the forefront of clean energy technologies [79]. Since their exploration in the early 2000s, CBNs have played a pivotal role in water electrolysis and steam methane reforming processes. Among recent innovations, graphene-based materials have emerged as particularly noteworthy materials. These materials, particularly when used as supports for metal nanoparticles, exhibit enhanced catalytic activities and stabilities. For instance, reduced graphene oxide (rGO) combined with CdS nanorods has been shown to boost hydrogen production nearly five-fold compared to pure CdS. This improvement is attributed to rGO’s ability of rGO to facilitate better charge separation and provide additional active sites. Carbon nanotubes (CNTs) have demonstrated substantial potential. Functionalized CNTs, particularly those treated with HCl and HNO_3_, act as hydrophilic and stable supports that enhance the dispersion and efficacy of the metal catalysts [80,81]. A carbon-based electrocatalyst has emerged as efficient HER catalyst with lower overpotentials and high durability. Another significant development in this field is the development of carbon-based single-atom catalysts (C-SACs) and the incorporation of non-noble metal or by non-doping into the carbon skeleton. Several transition metals like Cd, Zr, Nb, Y, V, etc., can be explored using the carbon-based catalyst, which can improve the electrocatalyst properties [82]. Advanced characterization techniques such as high-resolution transmission electron microscopy and density functional theory simulations have been instrumental in elucidating the atomic structures of these carbon-based electrocatalyst and uncovering the correlation between their structure and catalytic performance. Despite these advancements, several challenges remain. The stability of CBN catalysts under harsh reaction conditions, particularly in acidic environments, remains a concern. Moreover, the precise nature of the active sites in CBN catalysts continues to be debated, with some researchers emphasizing the role of metal–carbon interfaces and others focusing on carbon defects and doping. Addressing these complexities requires a deeper understanding of the fundamental properties and mechanisms underlying the catalytic behavior of these materials.

Thus, promising avenues for CBN research have emerged. The development of hierarchical carbon structures that integrate different carbon allotropes, such as by combining graphene with CNTs to leverage synergistic effects, is particularly exciting [83]. Furthermore, innovative synthesis techniques, such as atomic layer deposition, offer precise control over catalyst composition, potentially leading to enhanced performance. The exploration of hybrid materials that combine CBNs with other nanomaterials, such as metal–organic frameworks and two-dimensional transition metal dichalcogenides, also holds promise for achieving superior catalytic efficiency. CBNs present transformative opportunities to advance hydrogen-production technologies. Although substantial progress has been made, a considerable scope remains to optimize these materials, unravel their intricate catalytic mechanisms, and translate laboratory success into practical applications. With ongoing research, CBN catalysts are well positioned to play a critical role in the transition towards hydrogen-based energy.

#### 4.1.2. Metal Oxide Nanoparticles

Metal oxide nanoparticles have garnered significant attention, as both academia and industry have increasingly focused on cleaner energy solutions. Central to these efforts is the catalytic HER, in which metal oxide catalysts demonstrate considerable potential for generating hydrogen from water [84,85]. As the global demand for renewable energy continues to rise, the study and enhancement of metal oxide catalysts remains a focal point for achieving a sustainable energy future. The use of metal oxides for hydrogen production can be traced back to the 1973 oil crisis, a landmark event that spurred interest in the use of hydrogen as a clean energy carrier. Since then, extensive research has focused on the development of efficient and cost-effective metal oxide-based HER catalysts [86]. Transition metal oxides have garnered widespread attention owing to their natural abundance, structural stability, and favorable catalytic properties [87]. These characteristics make these materials promising candidates for hydrogen production.

In recent years, the field has expanded significantly with advancements in the catalytic performance of metal oxide nanoparticles, leading to innovative strategies for catalyst design and optimization. In addition to transition metal oxides, non-metal oxides have been explored, broadening the scope of materials and enabling novel approaches. Techniques such as nanostructuring, doping, and hybridization have proven to be particularly effective in enhancing the catalytic properties of metal oxide nanoparticles. Nanostructuring, for instance, increases the surface area and exposes more active sites, as observed for materials such as MgO, CaO, TiO_2_, ZnO, and ZrO_2_, which exhibit significantly higher activities than their bulk counterparts. Incorporating dopants or forming hybrid structures can further modify the electronic properties of the catalysts. Lin et al. [88] developed a catalyst for the selective conversion of bisphenol A (BPA) to hydrogenated bisphenol A (HBPA). BPA is widely used in industrial production but has potential harmful effects. In contrast, HBPA is regarded as a nontoxic and stable alternative. The researchers constructed a catalyst comprising highly dispersed ruthenium immobilized on nickel-modified aluminum oxide (Ru/Ni-Al_2_O_3_) and demonstrated that it effectively converted BPA to HBPA under mild conditions. The nickel promoter regulates the selectivity of the reaction. Various parameters, such as hydrogen pressure, reaction temperature, and liquid hourly space velocity (LHSV), also influence catalytic performance. The optimized catalyst achieved a BPA conversion rate of 100% and an HBPA selectivity of 96.4%. Furthermore, green synthesis methods for metal oxide nanoparticles have gained traction, emphasizing environmentally friendly approaches that not only reduce the ecological impact but also yield nanoparticles with enhanced catalytic properties. Recently, metal oxide perovskite nanostructures have been explored as photocatalyst and electrocatalyst materials for hydrogen production through water-splitting techniques. Bismuth titanate perovskite was prepared by a green synthesis method and utilized as photocatalyst for hydrogen production through a photochemical cell reactor and reported a hydrogen yield of 39.24 µmol/g obtained with methanol [89]. A-site cation nonstoichiometry perovskite Bax(Co,Fe,Zr,Y)O_3−δ_ has recently been studied as a electrocatalyst with a significant effect on the phase composition, which lead to enhanced lattice oxygen participation and interfacial interaction inside the nanostructures, promoting a catalytic performance for the OER in alkaline solutions [90]. Despite considerable progress, several challenges remain to be overcome. The long-term stability of metal oxide catalysts under harsh operational conditions and their scalability for industrial applications continue to pose significant obstacles. Moreover, the precise mechanisms underlying hydrogen evolution on metal oxide surfaces, particularly in alkaline media where water dissociation can become a limiting step, are still not fully understood. Addressing these challenges is critical for the successful deployment of metal oxide nanoparticle catalysts on a commercial scale.

Research in this domain is likely to prioritize rational design methodologies, the integration of in situ characterization techniques, and the development of multifunctional catalysts capable of supporting both hydrogen and oxygen evolution reactions. Sustainable synthesis methods will also remain a key focus, aiming to minimize the environmental impact while enhancing catalyst efficiency. The integration of advanced catalysts with renewable energy sources has the potential to create highly efficient and sustainable hydrogen-production systems. Through continued innovation and overcoming existing challenges, metal oxide nanoparticle catalysts have been poised to play a central role in the transition to a hydrogen-based economy.

### 4.2. Single-Atom Catalysts

SACs have recently emerged as significant advancements in materials science, offering the potential for hydrogen production. Unlike conventional catalysts, SACs consist of individual metal atoms dispersed on a supporting material, maximizing atom utilization, and presenting unique electronic properties that can be finely tuned to achieve optimal catalytic performance [91]. SACs enable researchers to exploit the potential of each metal atom, making these catalysts highly efficient and versatile for various reactions [80]. The concept of SACs was first introduced in 2011, marking a major milestone in heterogeneous catalysis. Compared to nanoparticle-based catalysts, SACs provide the maximum exposure of the active sites, achieving exceptional atomic efficiency [92]. The electronic structure of a single atom is influenced by its coordination environment, which allows the precise modulation of its catalytic properties [93,94]. This principle has led to rapid advancements in SAC technologies and their applications in catalysis. Recent progress in SAC development is particularly noteworthy for the HER, which is a critical step in hydrogen production. Scalable synthesis methods such as coprecipitation have shown significant promise. Chen et al. [95] discovered that fully exposed iridium clusters could be used as catalysts for the hydrogenation of N-heteroarenes, specifically quinoline. These catalysts exhibit higher activities and stabilities than single-atom and nanoparticle catalysts. Researchers have investigated the mechanism of the hydrogenation reaction on these catalysts and found that the unique geometric and electronic structures of the iridium clusters enable efficient hydrogenation via the Horiuti–Polanyi mechanism. In contrast, single-atom catalysts face difficulties in hydrogen activation and hydrogenation owing to their lack of metal–metal bonds and oxidized states. This study provides insight into the usefulness of fully exposed metal clusters in hydrogenation reactions and enhances our understanding of the reaction mechanism.

Another significant development in this field is the development of carbon-based single-atom catalysts (C-SACs). These materials, featuring individual metal atoms dispersed on carbon supports, optimize atomic efficiency and enable tailored electronic properties [80]. The choice of support material plays a key role in SAC performance. Carbon-based supports such as graphene and carbon nanotubes have been widely studied owing to their high conductivities and large surface areas [96]. C-SACs have shown remarkable performance in the oxygen reduction reaction (ORR), which is a critical process in hydrogen fuel-cell applications. Advanced characterization techniques such as high-resolution transmission electron microscopy and density functional theory simulations have been instrumental in elucidating the atomic structures of C-SACs and uncovering the correlation between their structure and catalytic performance. However, emerging research suggests that amorphous materials with unique surface structures can serve as superior supports. These materials offer unsaturated coordination sites that enhance single-atom anchoring and contribute to greater stability and activity. The adaptability of the support materials enables researchers to customize SACs for specific reactions and operating conditions. Despite these promising developments, several challenges remain to be overcome. A primary concern is the stability of single atoms under the reaction conditions, as atom aggregation can lead to cluster formation and can diminish catalytic efficiency. In addition, the precise nature of active sites in SACs remains unclear. While some studies attribute catalytic activity solely to the metal center, others highlight the critical role of the interaction between the metal atom and its coordination environment. These unresolved questions underscore the need for further investigation into SAC stability and the mechanisms underlying its catalytic properties. To advance SAC technology for practical hydrogen production, future research should prioritize key areas. The development of in situ characterization techniques to observe SAC behavior during reactions is essential, as is computational modeling to predict optimal electronic structures. Moreover, scalable synthesis methods that maintain high catalytic efficiency are critical for commercial adoption. Multifunctional SACs capable of catalyzing multiple reactions can further enhance the versatility and efficiency of catalytic systems. SACs represent an innovation with the potential to revolutionize clean energy technologies. By addressing the challenges associated with SAC stability, active-site characterization, and scalable synthesis, researchers can unlock its full potential. These advancements can transform hydrogen production and energy-storage solutions, significantly contributing to a sustainable energy future [97].

### 4.3. Bimetallic and Multi-Metallic Catalysts

**Figure 5:**
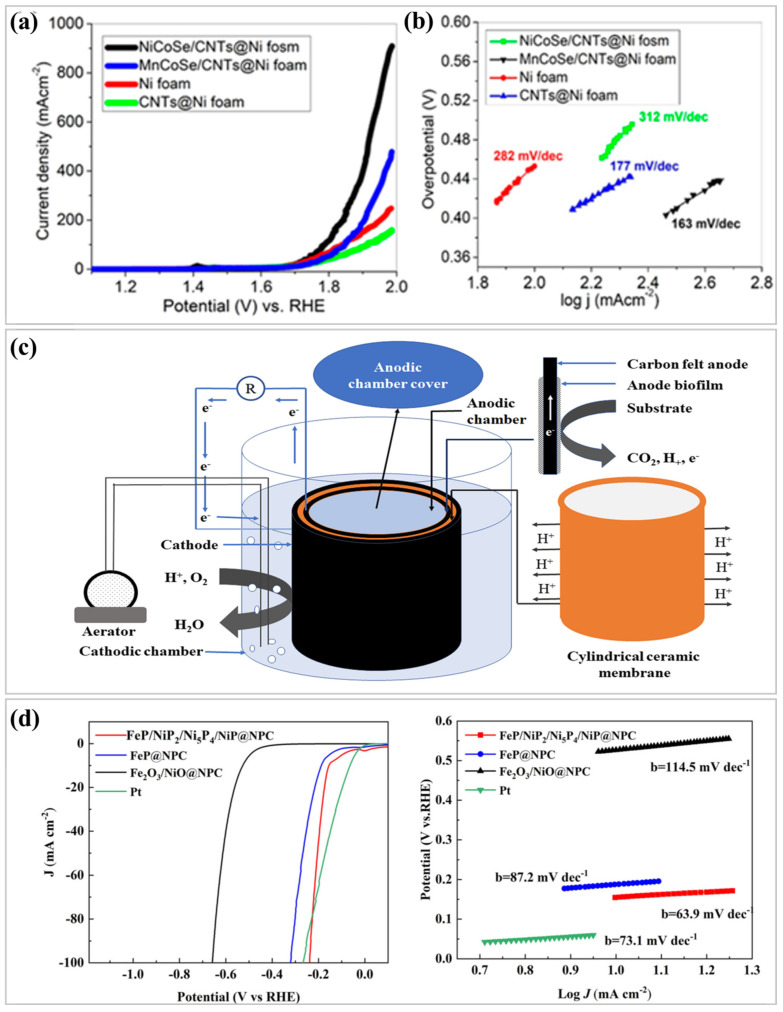
(**a**) Oxygen evolution reaction (OER) activity of Ni foam-based electrocatalysts and (**b**) Plot graphs of the unmodified and modified catalysts from the oxygen evolution reaction (OER) tests. [104], Copyright © 2024, MDPI. (**c**) Experimental setup and operation of two-chambered MFCs [105], Copyright © 2024, Springer-Nature. (**d**) Structural evolution and activity of Na-promoted Co-Fe bimetallic catalysts for CO_2_ conversion, reproduced with permission [106], Copyright © 2024, MDPI.

Bimetallic and multi-metallic catalysts have emerged as innovative materials for efficient hydrogen production, offering advantages in terms of catalytic performance and durability that monometallic catalysts often lack [98,99]. Although the use of multiple metals to build catalytic synergy has been explored for over a century, recent advancements in nanotechnology and material characterization have renewed the interest in these systems [100]. These developments have expanded their applicability to hydrogen production, marking a significant step forward in the field of catalysis. One prominent area in which bimetallic catalysts have demonstrated promise is methane steam reforming (MSR), a critical process for hydrogen generation. Incorporating Ni into noble or transition metals has consistently shown enhanced catalytic performance compared with traditional monometallic systems [101,102]. For instance, Fatesh et al. [103] studied the use of iron (Fe) as a promoter in nickel (Ni)-based catalysts supported by zirconia–alumina for the dry reforming of methane (DRM) to produce hydrogen (H_2_). The addition of Fe enhances the DRM performance of the catalysts by promoting reducible iron species and improving basicity. Catalysts with 1–4 wt.% Fe increased H_2_ yield, with a maximum of 87% at 3 wt.% Fe. The Fe-promoted catalysts exhibited excellent resistance to coke formation and maintained a high H_2_ yield because of the balanced rates of CH_4_ decomposition and carbon deposit diffusion. These results indicate that Fe-promoted zirconia–alumina-supported Ni catalysts are cost-effective and efficient systems for H_2_ production through DRM. In water electrolysis, bimetallic and multi-metallic catalysts enhance the efficiency of the HER and OER. A particularly innovative development involves bimetallic SACs, which utilize dual-metal atomic sites to enhance catalytic activity and stability while reducing material costs. Another promising application is ammonia decomposition, which has substantial potential for hydrogen production. Bimetallic catalysts have demonstrated remarkable efficiencies and stabilities in this domain. The synthesis of advanced electrocatalysts for energy conversion applications has garnered significant attention, as evidenced by recent research. For instance, Riaz et al. [104] explored the development of carbon nanotube composites incorporating bimetallic transition metal selenides as electrocatalysts for the oxygen evolution reaction (OER). Figure 5a shows the polarization curves of the Ni foam-based electrodes, highlighting the superior OER activity of NiCoSe/CNTs@NF with the lowest overpotential (0.560 V at 100 mAcm⁻^2^), while Figure 5b presents the Tafel slopes, confirming the enhanced charge transfer kinetics. These composites demonstrated remarkable performance, characterized by a low overpotential and improved charge transfer impedance. These attributes suggest their potential to enhance electrocatalytic efficiency, making them promising candidates for energy conversion and storage technologies. This study highlights the role of innovative material synthesis in addressing critical challenges in energy applications. In parallel, Jadhav et al. [105] investigated a novel tri-metal ferrite catalyst, Co_0.5_Cu_0.5_Bi_0.1_Fe_1.9_O_4_, tailored for the oxygen reduction reaction (ORR) in microbial fuel cells (MFCs). Schematic of the two-chambered MFCs in Figure 5c with a ceramic cylinder as the anodic chamber, a carbon felt anode, and a ceramic wall serving as a cation exchange membrane. When synthesized using a sol–gel auto-combustion process, the ferrite catalyst exhibited superior electrochemical performance compared to a bare carbon felt cathode, as demonstrated by the enhanced reduction current and lower charge transfer resistance. Notably, the MFC employing this catalyst achieved a power density of 11.44 W/m^3^ and a Coulombic efficiency of 21.4%, rivaling commercial Pt/C catalysts. This study underscores the potential of cost-effective alternatives, such as trimetal ferrites, to replace expensive platinum-based catalysts in sustainable energy systems. Zhang et al. [106] introduced a strategy in interface engineering to boost the catalytic activity of transition metal phosphides (TMPs) in the hydrogen evolution reaction (HER). Their multi-metallic phosphide nanoparticle catalyst, FeP/NiP_2_/Ni_5_P_4_/NiP, encapsulated in an N, P-doped carbon matrix, exhibited excellent performance in both acidic and alkaline environments. This success was attributed to the heterointerface between the different phosphides and the protective carbon matrix, which mitigated nanoparticle aggregation. Their findings presented a straightforward yet effective approach for designing robust catalysts for hydrogen production, paving the way for scalable clean energy solutions. Collectively, these studies emphasize the transformative potential of innovative materials and catalytic systems across diverse applications, from energy storage and microbial fuel cells to hydrogen evolution, fostering progress toward sustainable energy technologies.

Despite these advancements, several challenges must be addressed to realize the full potential of bimetallic and multi-metallic catalysts. Ensuring long-term stability under real-world conditions such as elevated temperatures and alkaline environments remains a critical hurdle. In addition, achieving precise control over atomic composition and metal distribution in multimetallic systems is technically complex. Issues such as metal segregation can compromise the catalytic performance, underscoring the need for refined characterization techniques and innovative design strategies. Future research will likely emphasize the development of earth-abundant metal combinations, enhanced scalability, and novel synthetic methods [99]. Bimetallic and multi-metallic catalysts represent key innovations in the advancement of hydrogen production technologies. By exploiting the synergistic interactions between different metals, these catalysts offer improved activities, selectivities, and durabilities. As research continues to refine their design and applications, these materials are expected to play a transformative role in promoting sustainable and efficient hydrogen production globally.

### 4.4. Metal–Organic Frameworks (MOFs)

MOFs have gained significant attention as catalysts for hydrogen production because of their high surface areas, tunable porosities, and flexible chemical compositions [107,108]. This section discusses recent advancements in MOF-based catalysts, highlighting breakthroughs, persistent challenges, and future directions, with a specific focus on water-splitting reactions, a key pathway in hydrogen generation. The exploration of MOFs as catalysts for hydrogen production began in the early 2000s, inspired by their established applications in gas storage and separation. Early studies focused on employing MOFs as supports for conventional catalysts such as platinum nanoparticles. However, as research progressed, the inherent catalytic properties of MOFs have attracted intense interest. Researchers have discovered that the metal nodes and organic linkers in MOFs can act as active sites for proton reduction, contributing directly to the HER [109]. The high surface area and porosity of MOFs further facilitate mass transport and ensure accessible active sites, whereas the structural tunability of MOFs allows precise control over their electronic and geometric properties, forming optimized catalytic centers [110,111].

**Figure 6:**
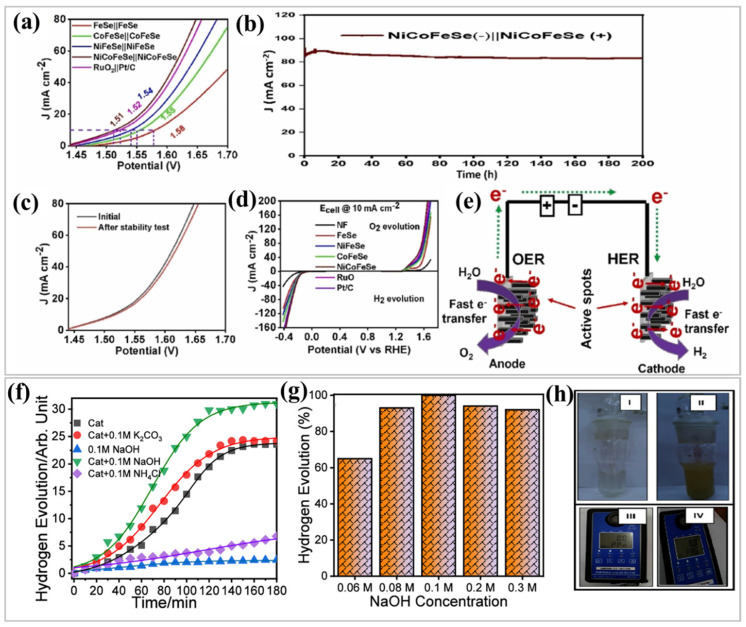
(**a**–**e**) Superior electrocatalytic performance and stability of NiCoFeSe for overall water splitting [112], Copyright © 2024, (**f**,**g**) effect of NaOH concentration on catalyst-free and catalyzed AB hydrolysis, and (**h**) Cu-MOF-74 effectively suppresses ammonia release during ammonia borane hydrolysis, as confirmed by Nessler’s reagent test and ammonia gas detection experiments (Figure **I**–**IV**). reproduced with permission [113], Copyright © 2024, Elsevier B.V.

A key advancement in this area has been the development of MOF-derived catalysts via controlled thermal treatment, which transforms MOFs into nanostructured metal/carbon composites with improved catalytic activity. Meshesha et al. [112] synthesized MOF-based Ni- and Co-doped iron selenide (NiCoFeSe) hexagonal nanorod electrocatalysts. Their research indicated that the NiCoFeSe catalyst exhibited exceptional performance in hydrogen evolution, oxygen evolution, and urea oxidation reactions, characterized by very low overpotentials. NiCoFeSe exhibited outstanding performance in overall water splitting, as demonstrated by its lower voltage requirements (Figure 6a), excellent stability in chronoamperometry tests (Figure 6b), sustained catalytic activity post-stability test (Figure 6c), half-cell voltage analysis (Figure 6d), and mechanistic illustration of electron movement (Figure 6e). These findings establish NiCoFeSe as a promising and durable electrocatalyst for efficient hydrogen production. They also developed a wireless flexible and rigid photovoltaic–electrochemical device utilizing NiCoFeSe as both the anode and cathode, achieving a solar-to-hydrogen efficiency of 11.1%. Additionally, they created an anion exchange membrane water electrolyzer incorporating NiCoFeSe as the anode and cathode, attaining a current density of 1.07 A cm^−2^ at 1.85 V, cell efficiency of 69.67%, and energy consumption of 47.85 kWh to produce 1 kg of hydrogen. Their findings demonstrated the potential of NiCoFeSe as an efficient and cost-effective material for water-splitting technologies.

Another significant innovation comes from bimetallic MOFs, in which the synergistic interaction between two metal centers enhances catalytic performance. For instance, Shingole et al. [113] examined the use of MOFs as catalysts for the hydrolysis of ammonia borane (AB) to produce hydrogen. The effect of NaOH concentration on AB hydrolysis was investigated in the presence and absence of Cu-MOF-74. (a) In the absence of the catalyst, NaOH alone exhibited a minimal catalytic effect. (b) With Cu-MOF-74, the addition of 0.1 M NaOH significantly enhanced hydrogen generation, indicating its role as a catalytic promoter. They synthesized a series of MOFs and identified Cu-MOF-74 as the most effective catalyst, exhibiting high turnover frequency and low activation energy. This study explored the influence of various factors, including ions, AB concentration, and catalyst concentration, on the AB hydrolysis reaction rate. Isotope labeling mass spectrometry techniques were employed to determine the source of hydrogen and clarify the reaction mechanism. Additionally, the study revealed that Cu-MOF-74 could capture ammonia during AB hydrolysis, indicating its potential application in fuel cells. Additionally, MOF-based heterostructures, such as MOF/graphene oxide composites, have shown improved charge transfer and enhanced active site exposure, further boosting HER activity [114,115]. Despite these advances, several critical challenges continue to affect the development of metal–organic framework-based catalysts. One major concern is stability, because many MOFs lack resilience under harsh acidic or alkaline conditions. This instability raises questions regarding whether the active catalytic species are due to the original MOF structure or whether they arise from the in situ formation of metal or metal oxide nanoparticles. Another challenge is the low electrical conductivity of most MOFs, which impedes electron transfer during electrocatalysis. Efforts to incorporate conductive additives or design inherently conductive MOFs are underway but remain in their early stages. Finally, scalability is a hurdle, because the synthesis of high-quality MOFs often requires costly precursors and complex procedures, making large-scale applications challenging. Table 3 highlights the unique advantages of each type of catalyst accompanied by notable nanomaterials for hydrogen production applications.

Several promising approaches have been investigated to address these challenges. One approach involves the rational design of active sites through computational studies, such as density functional theory (DFT) calculations, which can predict the optimal electronic structures and binding energies for HER catalysis. Another avenue is the use of advanced spectroscopic and microscopic techniques for in situ characterization, which allows researchers to better understand the true nature of the active sites and reaction mechanisms. Furthermore, the preparation of MOFs with hierarchical pore structures can improve the mass transport and active site accessibility, thereby enhancing the overall catalytic performance. Additionally, defect engineering or the intentional introduction of defects may open new active sites and improve catalytic activity. Finally, sustainable synthetic approaches, such as green chemistry methods and the use of earth-abundant materials, are essential to address scalability concerns and promote the widespread adoption of MOF-based catalysts. MOF-based catalysts hold substantial promise for hydrogen production and offer several advantages over traditional catalysts. However, challenges related to stability, conductivity, and scalability remain significant. Future research focusing on targeted design, in situ analysis, and sustainable synthesis may yield further breakthroughs in this field.

### 4.5. Practical Devices and AI-Supported Methods

Hydrogen has long been recognized as a clean fuel with immense potential; however, traditional production methods are hindered by high energy costs and carbon emissions. AI-driven catalyst design has enhanced efficiency, reduced reliance on expensive materials, and accelerated discovery. Artificial intelligence (AI) and machine learning (ML) have replaced trial-and-error approaches with predictive modeling, enabling the identification of efficient, earth-abundant catalysts [121]. By leveraging advanced computational techniques, such as density functional theory (DFT), AI improves catalyst performance predictions and facilitates biomimetic designs inspired by nature. In water electrolysis, AI has enhanced electrocatalysts by optimizing nanostructured materials to achieve superior activity, stability, and cost efficiency. Similarly, AI-assisted photocatalysis addresses efficiency limitations by refining semiconductor materials and doping strategies, making solar-driven hydrogen production more viable [21].

Despite these advancements, AI-driven catalyst design faces challenges such as inconsistent experimental datasets and the “black-box” nature of deep learning models. Additionally, many AI-predicted catalysts encounter difficulties with real-world scalability; however, emerging solutions such as quantum computing and cloud-based AI may help bridge this gap [122]. The future of AI-driven catalysis includes autonomous laboratories, AI-integrated quantum computing, and biomimetic catalysts that can replicate enzymatic hydrogen production. By refining computational models and integrating AI insights with experimental workflows, researchers can expedite the commercialization of AI-designed catalysts. AI is redefining hydrogen production by accelerating catalyst discovery and optimization, thereby making the process more efficient and sustainable. Overcoming challenges related to data quality and scalability is essential for realizing the full potential of AI. With continued advancements, AI is well-positioned to drive the global transition toward a hydrogen-powered future.

## 5. Challenges and Future Directions

This review provides a comprehensive overview of the latest developments in catalyst technology, with a focus on methods for enhancing hydrogen production. These findings underscore the pressing need for efficient and affordable catalysts to support the transition to sustainable energy sources. This need arises from the role of hydrogen as a clean energy carrier, which has great potential to meet global energy demands and address climate change challenges. Researchers have explored a wide range of catalytic materials with different compositions and properties. Noble metal catalysts, such as platinum, palladium, and rhodium, are highly active, but are limited by their high cost and scarcity. This review emphasizes innovations such as SACs and bimetallic catalysts, which promise to maintain efficiency while reducing the dependency on expensive metals. Transition metal catalysts are gaining attention owing to their abundance and versatility, with materials such as transition metal nitrides and molecular catalysts showing promising performance in the HER. Additionally, advancements in nanostructured catalysts, carbon-based nanomaterials, metal oxide nanoparticles, and MOFs have revealed unique properties that enhance their catalytic performance, marking a significant shift in catalyst design and application.

**Figure 7:**
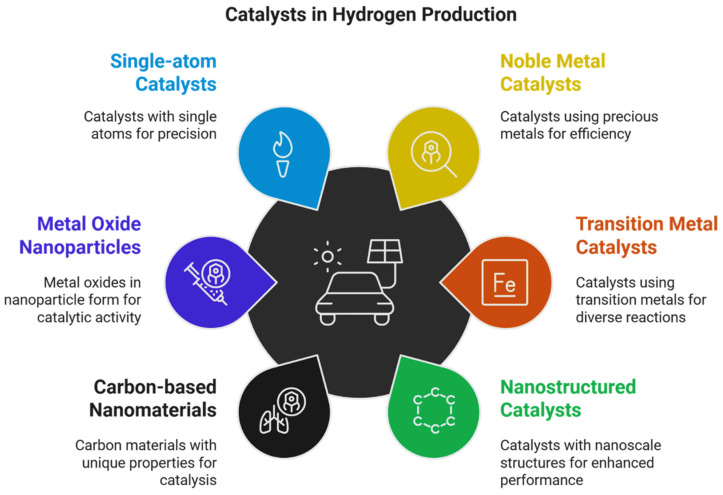
Overview of catalysts in hydrogen production.

However, despite these advancements, the development of catalysts for hydrogen production faces several challenges. For instance, scalability and cost-effectiveness remain significant obstacles because many high-performance catalysts involve complex synthesis methods that are difficult to apply on a large scale. Furthermore, the stability and longevity of these catalysts, particularly under acidic or alkaline conditions, continue to be critical issues that affect their practical application. A deeper understanding of the structure–function relationships within catalysts is necessary, as it can facilitate the design of more effective and reliable materials. These challenges highlight areas requiring further investigation to ensure that these catalysts can be utilized in real-world energy systems. This review identifies several promising research directions that could revolutionize hydrogen production. One key area is the development of multifunctional catalysts capable of performing multiple tasks, such as water splitting and CO_2_ reduction, to improve overall system efficiency. In addition, computational modeling and machine learning are expected to play essential roles in predicting the catalyst behavior, potentially accelerating the discovery of novel materials. Moreover, the integration of catalytic systems with renewable energy sources can significantly enhance the sustainability and scalability of hydrogen production, making it a viable alternative energy source. The broader implications of advancements in catalysis for hydrogen production extend beyond this specific application. By improving hydrogen production processes, these innovations could pave the way for a cleaner energy landscape that reduces reliance on fossil fuels and mitigates greenhouse gas emissions. Moreover, exploring diverse catalyst materials and innovative designs holds potential for breakthroughs in other catalytic applications across various industries. This interdisciplinary research effort, which bridges material science, engineering, and computational modeling, underlines the importance of collaboration in tackling global energy challenges and supporting sustainable solutions. A comprehensive visualization is presented in Figure 7, which shows various catalysts for hydrogen production, including single-atom, noble metal, metal oxide nanoparticles, transition metals, carbon-based nanomaterials, and nanostructured catalysts, each contributing unique properties to efficient and precise catalytic activity.

Although these advancements are promising, several research gaps remain and warrant further investigation. Understanding the long-term stability of catalysts under real-world conditions is essential, particularly for transition metal- and carbon-based catalysts. The development of scalable and cost-effective synthesis methods for high-performance catalysts is crucial for facilitating their widespread use in industrial applications. Additionally, advanced in situ characterization techniques are required to study the dynamic behavior of catalysts during reactions, which would enable more targeted design improvements. The environmental impact of the catalyst production is also a key consideration, emphasizing the need for eco-friendly and earth-abundant materials for future research. Finally, integrating these catalytic systems with renewable energy sources remains a critical avenue for optimizing hydrogen production in response to intermittent energy supplies. The field of catalysis for hydrogen production is rapidly advancing and offering hope for a sustainable energy future. Ongoing interdisciplinary collaboration and increased research investment are vital for overcoming current challenges and exploring new possibilities in this transformative area. These efforts will play a central role in making hydrogen production both viable and sustainable, thus contributing to the broader goal of clean and resilient energy infrastructure.

## Figures and Tables

**Table 1:** Key performance indicators (KPIs) related to hydrogen evolution mechanisms and catalytic performance.

Key Indicator	Specific Metrics	Enhancement Strategies	Ref.
Activity	Overpotential Tafel slope Current density	Catalyst tailoring Heterostructure engineering	[48]
Stability	Cycle retention Time durability	Doping Surface engineering Robust material selection	[49]
Selectivity	Selective hydrogen production Minimal byproducts	Modulating electronic structures Optimized synthesis techniques	[50]
Structural Design	Porosity Active surface area Interface stability	Heterostructure and interface engineering Defect creation	[51]
Scalability	Cost-effectiveness Material availability	Use of earth-abundant materials Green synthesis methods	[52]
Electron Transfer	Charge transfer resistance Electronic conductivity	Metal–support interactions Doping	[53]
Hydrogen Adsorption	Gibbs free energy (ΔGH) Adsorption kinetics	Electronic structure tuning Transition metal catalysts	[51]
Sustainability	Environmental impact Renewability	Use of waste or renewable materials Energy-efficient processing	[52]
Interfacial Engineering	Active site density Defect-induced activity	Creating heterointerfaces Defect engineering	[50]

**Table 2:** Key properties of materials for hydrogen production catalysts.

Nanomaterials	Physical Properties	Catalytic Properties	Hydrogen Interaction Properties	Ref.
Carbon-based	-High surface area -Good thermal stability -Electrical conductivity	-Active sites on surface -High catalytic efficiency -Low overpotential	High hydrogen adsorption, catalytic efficiency in electrochemical reactions	[73]
Metal oxide	-High surface area -Thermal stability -Mechanical strength	-High TOF -Catalytic stability -Reduction in activation energy	Efficient hydrogen storage and catalytic activity in water splitting	[74]
Single-atom	-Supported on high-surface-area materials like porous carbons -Thermal stability	-Maximized TOF due to isolated active sites -Low overpotential	High hydrogen adsorption/desorption efficiency, excellent for water splitting	[75]
Bimetallic/multi-metallic	Enhanced mechanical and thermal strength through alloying	-Synergistic catalytic effects -Reduced activation energy -Stable performance	Enhanced hydrogen adsorption and storage, high catalytic turnover in hydrogen evolution reactions	[76]
MOFs	Large surface area, tunable pore size, thermal stability	-High catalytic stability -Efficient reduction of overpotentials -Tunable active sites	High hydrogen storage capacity, catalytic efficiency in water splitting, and other redox reactions	[77]

**Table 3:** Performance Comparison of Hydrogen Production Catalysts with Specific Nanomaterials.

Catalyst Type	Nanomaterial Examples	Activity	Stability	Selectivity	Overpotential	Cost-Effectiveness	Surface Area and Active Sites	Reference
Carbon-based nanomaterials	Graphene, graphene oxide (GO), carbon nanotubes (CNTs), carbon dots (CDs)	High ORR performance, superior to Pt in alkaline solutions	Excellent in alkaline media	Comparable to Pt in methanol tolerance	Moderate (~300 mV at 10 mA/cm^2^)	Low-cost, non-precious materials	High surface area, large pore channels	[116]
Metal oxide nanoparticles	Co_3_O_4_, Fe_2_O_3_, MnO_2_, NiO	Superior for OER with 270 mV at 10 mA/cm^2^	High durability in alkaline solutions	High due to structural optimization	Moderate (270 mV)	Cost-effective	Moderate surface area, enhanced by doping	[117]
Single-atom catalysts	Pt on SnS_2_, Co on N-doped graphene	Comparable to 10 wt% Pt/C in HER	Ultrahigh stability, long-term durability	High due to isolated active sites	Very low (~51 mV at 10 mA/cm^2^)	Moderate	Maximal utilization of atoms in intercalated structures	[118]
Bimetallic catalysts	Ni-Co, Fe-Co, Pt-Ni	~5× improvement over Co@C monometallic	Superior under mild conditions	Excellent chemoselectivity	Low (~41.7 mV dec⁻^1^)	High for non-noble catalysts	Large active sites due to bimetallic synergy	[119]
MOFs	ZIF-8, UiO-66, MIL-101	Highly active with ultralow overpotential	High stability, resistant to deactivation	Selective to desired products	Low (~286 mV at 10 mA/cm^2^)	Moderate	Exceptional surface area, diverse active sites	[120]
